# Predicting developmental outcomes in premature infants by term equivalent MRI: systematic review and meta-analysis

**DOI:** 10.1186/s13643-015-0058-7

**Published:** 2015-05-17

**Authors:** Janneke van’t Hooft, Johanna H. van der Lee, Brent C. Opmeer, Cornelieke S. H. Aarnoudse-Moens, Arnold G. E. Leenders, Ben Willem J. Mol, Timo R. de Haan

**Affiliations:** Department of Obstetrics and Gynecology, Academic Medical Center, PO Box 22660, 1100 DD Amsterdam, The Netherlands; Pediatric Clinical Research Office, Emma Children’s Hospital, Academic Medical Center, PO Box 22660, 1100 DD Amsterdam, The Netherlands; Clinical Research Unit, Academic Medical Center, PO Box 22660, 1100 DD Amsterdam, The Netherlands; Pediatric Psychosocial Department, Emma Children’s Hospital, Academic Medical Center, PO Box 22660, 1100 DD Amsterdam, The Netherlands; Medical Library, Academic Medical Center, PO Box 22660, 1100 DD Amsterdam, The Netherlands; Robinson Research Institute, School of Pediatrics and Reproductive Health, University of Adelaide, Adelaide, 5000 SA Australia; Department of Neonatology (H3-147), Emma Children’s Hospital, Academical Medical Center, PO Box 22660, 1100 DD Amsterdam, The Netherlands

**Keywords:** Premature, Preterm, Development, White matter, MRI

## Abstract

**Background:**

This study aims to determine the prognostic accuracy of term MRI in very preterm born (≤32 weeks) or low-birth-weight (≤1500 g) infants for long-term (>18 months) developmental outcomes.

**Methods:**

We performed a systematic review searching Central, Medline, Embase, and PsycInfo. Two independent reviewers performed study selection, data extraction, and quality assessment. We documented sensitivity and specificity for three different MRI findings (white matter abnormalities (WMA), brain abnormality (BA), and diffuse excessive high signal intensity (DEHSI)), related to developmental outcomes including cerebral palsy (CP), visual and/or hearing problems, motor, neurocognitive, and behavioral function. Using bivariate meta-analysis, we estimated pooled sensitivity and specificity and plotted summary receiver operating characteristic (sROC) curves for different cut-offs of MRI.

**Results:**

We included 20 papers published between 2000 and 2013. Quality of included studies varied. Pooled sensitivity and specificity values (95 % confidence interval (CI)) for prediction of CP combining the three different MRI findings (using normal/mild vs. moderate/severe cut-off) were 77 % (53 to 91 %) and 79 % (51 to 93 %), respectively. For prediction of motor function, the values were 72 % (52 to 86 %) and 62 % (29 to 87 %), respectively. Prognostic accuracy for visual and/or hearing problems, neurocognitive, and/or behavioral function was poor. sROC curves of the individual MRI findings showed that presence of WMA provided the best prognostic accuracy whereas DEHSI did not show any potential prognostic accuracy.

**Conclusions:**

This study shows that presence of moderate/severe WMA on MRI around term equivalent age can predict CP and motor function in very preterm or low-birth-weight infants with moderate sensitivity and specificity. Its ability to predict other long-term outcomes such as neurocognitive and behavioral impairments is limited. Also, other white matter related tests as BA and DEHSI demonstrated limited prognostic value.

**Systematic review registration:**

PROSPERO CRD42013006362

**Electronic supplementary material:**

The online version of this article (doi:10.1186/s13643-015-0058-7) contains supplementary material, which is available to authorized users.

## Background

Preterm birth is associated with an increased risk of neurodevelopmental problems [1]. Magnetic resonance imaging (MRI) is increasingly being used to identify cerebral white matter lesions in the brain of preterm infants at term equivalent age. It is claimed to be a valuable tool to predict neurodevelopmental outcomes in very preterm infants, and its clinical use is, therefore, being promoted [2, 3]. However, the prognostic accuracy of white matter related MRI abnormalities for long-term developmental outcomes is debatable and its use as a standard of care is not yet recommended by the American Academy of Neurology Quality Standards [4]. The lack of meta-analytic synthesis of the primary studies reporting prognostic values, which tends to show conflicting results, hampers the debate.

Subsequently, the lack of knowledge about the prognostic accuracy of term MRI hampers an adequate interpretation of this test. This may invoke unwanted effects, as parents may worry unnecessarily about the possible abnormal development of their child [5, 6]. However, if term MRI can predict neurodevelopmental outcomes accurately, the use of this expensive diagnostic procedure as part of standard care could be justified as it may select high risk infants for prolonged and intensive supportive care.

Our study aims to evaluate the following two questions:

1. What is the prognostic accuracy (in terms of sensitivity and specificity) of white matter related abnormalities seen on term MRI for long-term developmental outcomes of infants born very preterm or with low birth weight?

2. Is there a difference in prognostic accuracy between the three types of white matter abnormalities as seen on term MRI including white matter abnormality, a combination of cerebral white matter lesions defined as “brain abnormality,” and diffuse excessive high signal intensity? To answer these questions, we performed a systematic review and meta-analysis on the subject.

## Methods

We performed a systematic review following the guidance of the PRISMA statement, Cochrane Handbook for Systematic Reviews of Diagnostic Test Accuracy and other recommendations found in the literature [7–9], with a prospectively published protocol at the Prospero database (www.crd.york.ac.uk/PROSPERO/display_record.asp?ID=CRD42013006362#.VVMAX47tlBc).

### Search strategy

We searched Central, Medline, Embase, and PsycInfo from their inception to November 2013 for relevant studies. The search was performed by a trained clinical librarian (AL) and two other authors (TdH and JvH). Broad text and MeSH terms were used. Also, keywords of eligible papers were screened and included in the final search. We did not apply any language restrictions. The search was limited to studies including humans. The full search in all these databases can be seen in Additional file 1. References from included studies were checked. Abstracts and reports from meetings were included only if they related directly to previously published work.

### Eligibility criteria

The following inclusion criteria were used to select studies: (1) the study pertained to infants born at a gestational age ≤ 32 weeks and/or birth weight ≤ 1500 g; (2) MRI should be planned at term equivalent age (37–42 weeks) with a maximum range of 3 weeks earlier or later (34–45 weeks); (3) MRI findings should be related to any developmental outcome; and (4) developmental follow-up should be performed ≥18 months postnatal age. Isolated single case studies and review articles were not included.

Abstracts were screened for eligibility by two independent reviewers (JvH and TdH). Full-text articles were retrieved if applicable to the core research question, or if the abstract did not supply sufficient information. Any disagreement was set by discussion until consensus. The same two reviewers appraised the methodological quality and performed the data extraction. Any disagreement at this stage was resolved by a third reviewer.

### Methodological quality

Due to lack of existing quality assessment tools for prognostic accuracy studies, we developed a modified version of the QUADAS-2 assessment tool [10] to evaluate the risk of bias (see Additional file 2).

### Data extraction

A standardized data extraction form (see Additional file 3) was used to record study information. The results of white matter abnormalities (WMA) and brain abnormalities (BA) are usually expressed as either no, mild, moderate, or severe abnormalities as described by Inder and Woodward et al. [11, 12]. Where possible we defined two cut-offs, i.e., (1) no abnormality vs. mild, moderate, or severe abnormality, reported as “normal vs. any” and (2) no or mild abnormality vs. moderate to severe abnormality, reported as ‘normal/mild vs. moderate/severe’. BA was defined as a combination of WMA plus presence of other brain abnormalities such as ventricular hemorrhage or increased ventricle size. For diffuse excessive high signal intensity (DEHSI), the results are usually expressed as either present or absent. Therefore, only one cut-off was used in the 2 × 2 tables presenting the results for these MRI findings.

The cut-off point for unfavorable developmental outcome was defined as a minus 2 standard deviations (−2SD) difference from the mean for each MRI finding. If this cut-off was not reported (but for example, only a −1.25 or −1SD), we used the reported cut-off in the meta-analysis.

In cases of duplicate reporting, i.e., the same cohort was described in two papers or one paper reporting developmental outcomes at different time points of age, we used data from the paper that reported the developmental outcome at a comparable age with the other included papers. For example: if two papers reported motor skills at 2 years of age and one paper reported at 2 and 6 years of age, the reporting at 2 years of age was used. In case two papers reported the same cohort at similar ages, the study with the largest sample size and least quality concerns was selected. If the required data could not be extracted from the publication, authors were contacted by email. All data were entered in Review Manager (RevMan) version 5.3. Copenhagen: The Nordic Cochrane Centre, The Cochrane Collaboration, 2012.

### Statistical analysis

We performed a meta-analysis using a bivariate modeling approach [13]. In view of the observed heterogeneity, a random-effects model was used. We compared pooled sensitivity and specificity (95 % confidence intervals); likelihood ratios of positive and negative test results (LR+/LR−) were calculated from the pooled sensitivity and specificity; diagnostic odds ratios (DOR), and posttest probabilities of three different MRI findings (WMA, BA and DEHSI), for all types of developmental outcomes. Sensitivity and specificity for individual studies and summary receiver operating characteristic curves (sROC) were plotted to visualize possible heterogeneity of data and overall test accuracy.

## Results

**Fig. 1:**
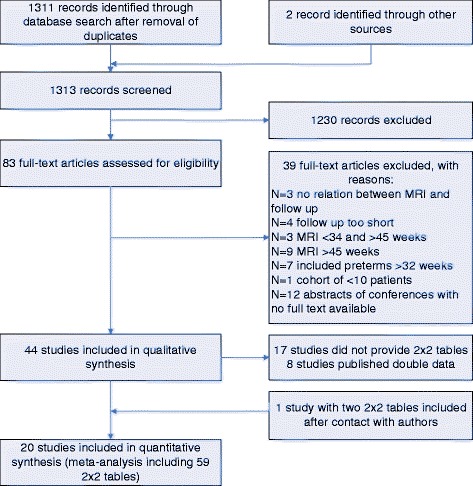
Flowchart of study selection

Our search strategy yielded 1311 citations after removal of duplicates (Fig. 1). A total of 44 papers met the inclusion criteria, of which 27 papers provided 2 × 2 tables. One more relevant paper was identified by contact with the authors. After excluding multiple publications from the same cohorts (8 papers), a total of 20 papers were available for the meta-analysis.

The 20 papers were all published between 2000 and 2013. These papers reported on 12 different cohort studies (2 retrospective and 10 prospective) including 1287 patients (682 male and 605 female). The extracted data provided 54 2 × 2 tables for WMA, BA, or DEHSI. These three MRI findings were used for the prediction of various developmental outcomes: cerebral palsy (CP), visual and/or hearing problems, motor, neurocognitive, and behavioral function, as well as a combination of problems in these domains defined as ‘neurodevelopmental impairment’ (NDI). Study characteristics are shown in Additional file 4: Table S1.

Studies from which 2 × 2 tables could not be derived (*n* = 17 papers, not reported in this manuscript) reported continuous data with no cut-offs. These studies mostly reported the following MRI tests: cerebellar abnormalities, volumes, and diameter measures of the brain (total brain or specific regions as hippocampus, corpus callosum, or ventricles).

### Methodological quality of included studies

**Fig. 2:**
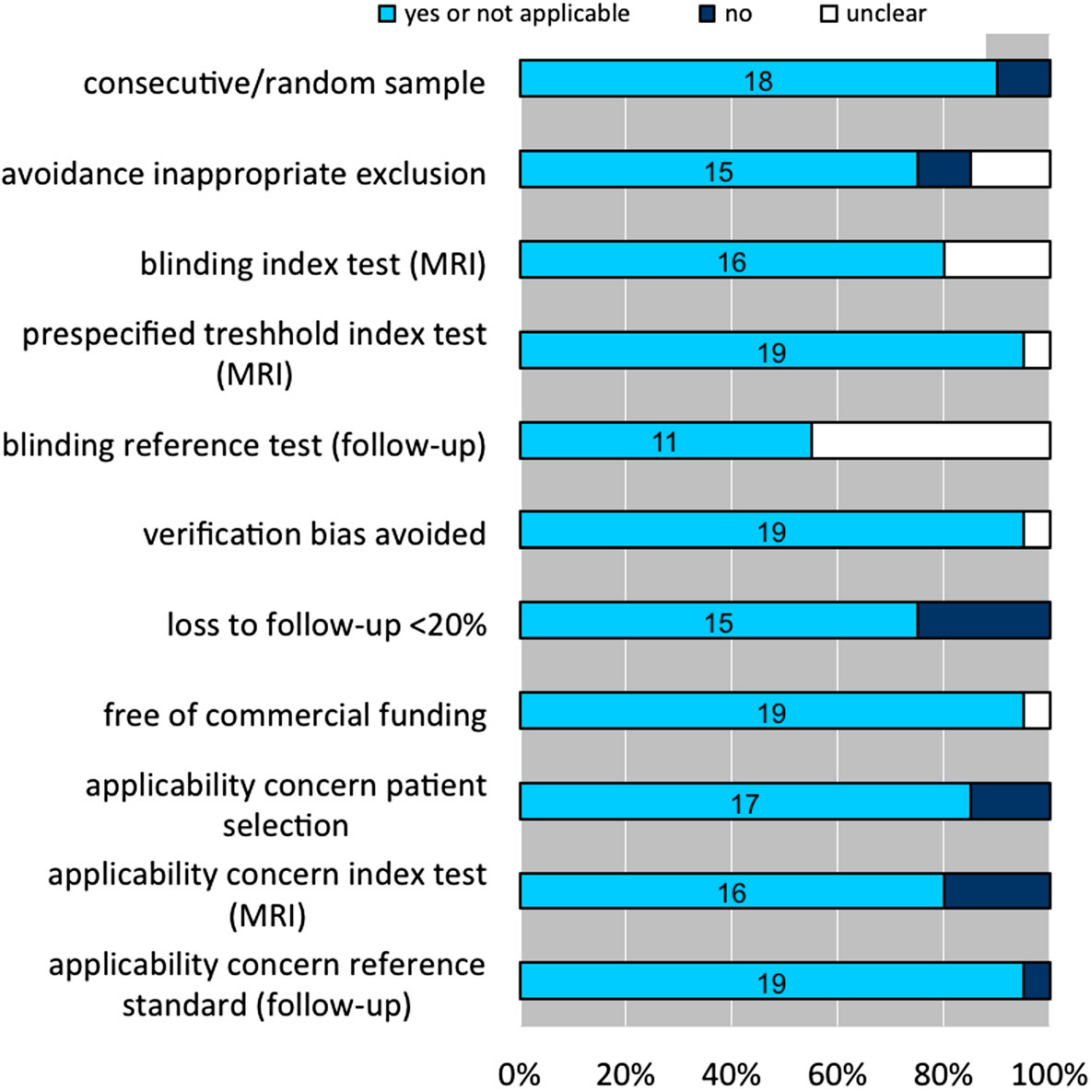
Quality assessment of included studies in meta-analysis (*n* = 20)

In general, 70 to 90 % of the included studies scored positive on each of the QUADAS-2 quality assessment items (Fig. 2). For example, 90 % of the studies included in the meta-analysis used a consecutive sample of very preterm born and/or low-birth-weight neonates over a specific period of time in their clinic (Fig. 2). In general, a good description of the MRI test and reference standard was provided, as well as a verification process to all neonates who had a MRI performed. However, almost 50 % of the papers did not report blinding of the test results, i.e., results of the MRI findings are not (made) available to the person performing the follow-up neurodevelopmental test.

### Meta-analysis

**Fig. 3:**
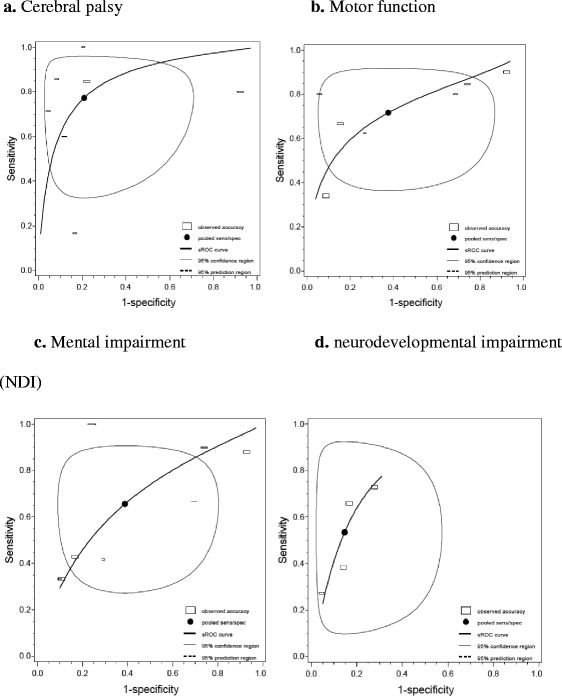
Pooled sensitivity and specificity with sROC reporting four developmental outcomes detected by any MRI abnormality (including white matter abnormality, brain abnormality or diffuse excessive high signal intensity using ‘normal/mild vs. moderate/severe’ cut-off). **a** (*n* = seven studies): pooled sensitivity 77 % (53 to 91 %) and specificity 79 % (51 to 93 %). **b** (*n* = seven studies): pooled sensitivity 72 % (52 to 86 %) and specificity 62 % (29 to 87 %). **c** (*n* = seven studies): pooled sensitivity 66 % (41 to 84 %) and specificity 53 % (35 to 71 %). **d** (*n* = four studies): pooled sensitivity 61 % (34 to 83 %) and specificity 85 % (75 to 92 %). The individual studies are visualized as *squares* with the *horizontal axis* corresponding to the total non-diseased neonates and *vertical axis* the total diseased neonates of that particular study population, i.e., a *flat square* represents a low prevalence of the disease, and the *surface of the square* represents the size of the study population

The reported sensitivity and specificity were generally higher for the WMA tests when compared to BA or DEHSI findings (Table 1). Fig. 3 shows the sROC curves for prediction of four different developmental delays related to any MRI abnormality (combination of WMA, BA, or DEHSI tests) using a ‘normal/mild vs. moderate/severe’ cut-off. The sROC curve for prediction of CP shows a curve that lies the most towards the (optimal) upper left corner of the ROC space. Also the sROC curve for prediction of motor function has a tendency to the upper left corner. The sROC curves for mental impairment and neurodevelopmental impairment, which are visualized in Fig. 3, are heading more towards the diagonal (non-discriminating) line of the ROC space.

**Table 1:** Results from bivariate analysis on sensitivity (Sens), specificity (Spec), 95 % confidence interval (95 % CI), diagnostic odds ratio (DOR), positive/negative likelihood ratio (LR+ and LR−), and pretest and posttest probabilities

MRI test with used cut-off	Developmental outcome	No. of studies	No. of neonates	Sens (95 % CI) %	Spec (95 % CI) %	DOR	LR+	LR−	Pretest probability (%)	Posttest probability	
										Positive	Negative
WMA—“normal vs. any”	CP	1 [35]	125	100 (70–100)	81 (73–87)	>100	5.27	<0.01	7.2	29.0	<0.01
	IQ	2 [36, 37]	283	79 (65–88)	41 (18–69)	2.61	1.34	0.51	16.6	21.1	9.2
	Language	2 [37, 38]	283	87 (69–97)	30 (23–39)	2.78	1.24	0.44	5.3	6.5	2.4
	Mental development	3 [12, 35, 39]	448	81 (59–93)	49 (26–73)	4.13	1.60	0.39	13.8	20.4	5.9
	Motor	3 [12, 35, 40]	485	87 (74–94)	51 (26–76)	7.29	1.80	0.25	17.3	27.4	5.0
	Vision/hearing	2 [12, 35]	125	62 (13–95)	53 (23–82)	1.88	1.33	0.71	31.2	37.6	24.4
WMA—“normal/mild vs. moderate/severe”	CP	2 [41, 42]	164	67 (38–87)	92 (85–96)	22.35	8.11	0.36	6.7	36.8	2.5
	IQ	2 [36, 37]	283	53 (39–67)	83 (77–87)	5.41	3.06	0.57	16.6	37.9	10.2
	Language	2 [37, 38]	283	47 (24–71)	86 (82–90)	5.46	3.38	0.62	5.3	15.9	3.4
	Mental development	3 [12, 39, 41]	398	38 (26–52)	87 (83–91)	4.21	2.98	0.71	13.8	32.3	10.2
	Working memory	2 [43, 44]	258	24 (17–32)	88 (78–94)	2.26	1.96	0.87	47.7	64.1	44.2
	Motor	3 [12, 40, 41]	435	54 (30–77)	90 (84–94)	10.59	5.37	0.51	17.5	53.2	9.7
	NDI	1 [12]	167	38 (26–53)	86 (78–91)	5.28	2.70	0.72	28.1	51.4	22.0
BA—“normal vs. any”	CP	2 [36, 45]	277	90 (68–98)	60 (54–66)	13.71	2.27	0.17	7.2	15.0	1.3
	Mental development	1 [46]	180	100 (61–100)	60 (52–67)	>100	2.49	0.00	3.3	7.5	<0.01
	Behavior	1 [47]	177	76 (61–88)	37 (29–46)	1.88	1.21	0.64	23.7	27.3	16.6
	Hearing	2 [36, 46]	397	100 (51–100^a^)	58 (53–63)	>100	2.49	0.00	2.0	4.9	<0.01
	NDI	2 [36, 46]	424	81 (69–89)	68 (61–75)	9.44	2.57	0.27	13.9	29.3	4.2
BA—“normal/mild vs. moderate/severe”	CP	3 [46, 48, 49]	273	90 (74–97)	80 (75–85)	37.83	4.56	0.12	11.4	36.9	0.02
	Mental development	2 [46, 50]	216	82 (8–100)	75 (69–81)	13.80	3.31	0.24	8.3	23.1	0.02
	Hearing	2 [36, 46]	397	100 (51–100^a^)	75 (70–79)	>100	3.97	<0.01	2.0	7.5	<0.01
	Motor	1 [50]	34	63 (31–86)	73 (54–86^a^)	4.53	2.32	0.51	23.5	41.7	13.6
	Behavior	1 [47]	177	33 (20–50)	78 (70–84)	1.75	1.50	0.86	23.7	31.8	21.1
	NDI	3 [36, 51, 52]	405	60 (39–78)	86 (70–94)	8.96	4.17	0.47	28.2	54.0	11.7
DEHSI	CP	2 [41, 45]	421	46 (9–89)	39 (3–92)	0.55	0.76	1.38	6.2	4.8	8.3
	Mental development	3 [35, 41, 53]	362	87 (72–94)	19 (9–35)	1.53	1.07	0.70	10.5	11.1	7.6
	Motor	3 [35, 41, 53]	362	86 (70–94)	20 (8–40)	1.48	1.07	0.72	10.5	11.1	7.8

If bivariate model could not estimate 95 % CI for pooled sensitivity for two studies, estimate is based on study with largest sample size

*BA* brain abnormality, *DEHSI* diffuse excessive high signal intensity, *NDI* neurodevelopmental impairment, *WMA* white matter abnormality

^a^data derived after contact with author

The pooled sensitivity and specificity values (95 % confidence interval (CI)) for prediction of CP were 77 % (53 to 91 %) and 79 % (51 to 93 %), respectively. Almost similar values were found for the prediction of motor function with a sensitivity of 72 % (52 to 86 %) and specificity of 62 % (29 to 87 %). Lower values were found for mental development and NDI with sensitivity of 66 % (41 to 84 %) and 53 % (35 to 71 %), respectively, and specificity of 61 % (34 to 83 %) and 85 % (75 to 92 %). Using a “normal vs. any” cut-off, pooled sensitivity and specificity values were 84 % (45 to 97 %) and 58 % (27 to 84 %) for prediction of CP; 76 % (48 to 92 %) and 26 % (8 to 57 %) for prediction of motor function; and 85 % (74 to 92 %) and 36 % (20 to 56 %) for prediction of mental development, respectively.

**Fig. 4:**
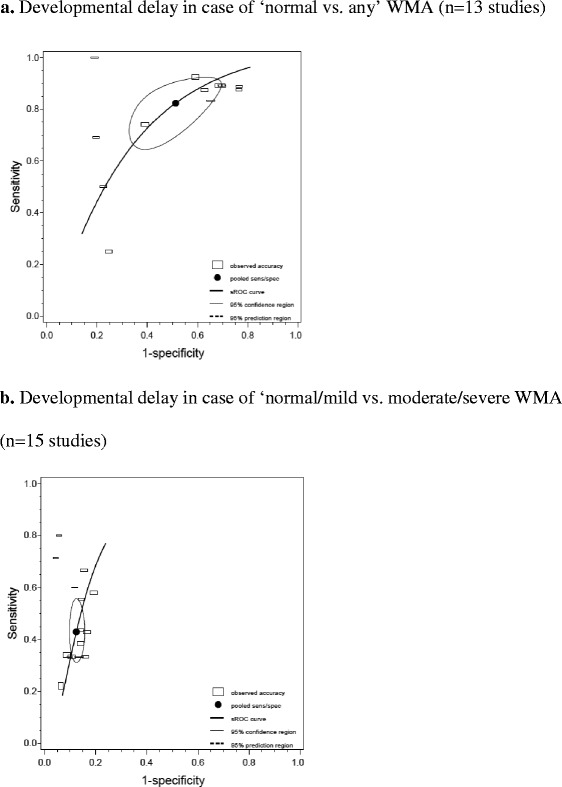
Pooled sensitivity and specificity with sROC corresponding to two different cut-offs of WMA for prediction of for various developmental outcomes/delays (cerebral palsy, IQ, working memory, visual and/or hearing, mental development, language and motor function delay). **a** Developmental delay in case of “normal vs. any” WMA (*n* = 13 studies). **b** Developmental delay in case of “normal/mild vs. moderate/severe” WMA (*n* = 15 studies). The *line* represents the sROC curve. The *black dot* represents the pooled sensitivity and specificity. The *blank squares* represents the individual studies, with the *horizontal axis* corresponding to the total non-diseased and *vertical axis* the total diseased of that particular study population

Figure 4 shows the sROC curves corresponding to the two different cut-offs: “normal vs. any” and “normal/mild vs. moderate/severe” when only the results of WMA are taken into consideration for prediction of various developmental outcomes. If only moderate to severe WMA lesions are coded as abnormal (“normal/mild vs. moderate/severe”), the specificity increases and the sensitivity decreases.

The spread of the individual studies alongside the sROC curves in Figs. 3 and 4 shows a substantial heterogeneity of the collected data explained by a threshold effect. The threshold effect is similar to the shift in sensitivity and specificity as described above, yet without an explicit change in cut-off levels. The shift is presumably the result of an implicit use of a different threshold, e.g., following from subjective judgments or calibration of diagnostic devices.

## Discussion

This study shows that the presence of moderate/severe WMA on MRI performed around term equivalent age can predict CP and motor function in very preterm or low-birth-weight neonates with moderate sensitivity and specificity. The ability to predict other long-term outcomes such as neurocognitive and behavioral impairments is limited. Also, other white matter related tests as BA and DEHSI demonstrated limited to no prognostic value.

In the last decade, the use of MRI as a screening tool for very preterm and low-birth-weight neonates has been a topic of major interest and several reviews have been published on its use [3, 14–18]. Most of these reviews are narrative (describing practical issues like sedation for MRI and/or different types of MRI techniques) or examined the impact of preterm birth and brain abnormalities on long-term development through the use of MRI. Although none of them systematically reported test accuracy of MRI for prediction of developmental outcome, most of these reviews, however, recommended the use of term MRI in clinical practice. To our best knowledge, our study is the first that systematically reviews the prognostic accuracy of different MRI findings on various long-term developmental outcomes.

### Clinical implications

The data in our meta-analysis suggest that presence of moderate/severe WMA has higher positive likelihood ratio, and absence of any WMA has a higher negative likelihood ratio than any other tests that we now use for preterm infants (e.g., cranial ultrasound or neurological examination) [19]. The prognostic accuracy of WMA finding on MRI therefore supports the use of MRI for preterm infants. However, whether this alters clinical management is a different question. Answering this question was beyond the scope of our meta-analysis. In our opinion however, showing potential prognostic accuracy of a test does not directly justify its clinical use as a standard test. The usefulness of this tool for clinical decision-making requires the presence of possible treatment or specified follow-up strategies following the results of the MRI [20]. At present, there is no specific treatment available addressing the needs of infants with abnormal white matter on MRI. However, the use of term MRI results may give focus to specific follow-up programs (i.e., offering a screening tool for developmental disorders at an earlier age) or improve selection of neonates for early intervention programs (i.e., physiotherapy or speech therapy). Also, available MRI results may help parents of prematurely born infants to better prepare for the future.

On the other hand, after screening all very preterm born or low-birth-weight neonates with a term MRI, there is no other tests available with better accuracy. Therefore, the possible harm due to false positive and false negative results must be taken into consideration. The value of being timely informed (value of information) must be weighted against the possibility of unnecessary concern for adverse outcome [21, 22]. For example, based on the results of this meta-analysis, we can expect that the finding of moderate to severe WMA in a very preterm born child will increase the probability of developing CP from the known prevalence of 7 % in this population to 37 % (Table 1). This raises the question if this increase in probability will change practice for both the clinician and patient. More specifically, will the clinician offer a different follow-up program when the risk of developing CP is 37 % instead of 7 %? And will the negative posttest probability of 2.5 % (i.e., 2.5 % will still develop CP after a normal MRI test result) justify a denial of follow-up to those with normal MRI?

Our meta-analysis also shows that adverse outcomes, such as neurocognitive and behavioral impairments, could not be predicted by term MRI abnormalities. Compromised white matter may result in more “subtle” impairments in such areas of the child’s long-term function. The limited prognostic value of WMA for these specific outcomes also suggests that despite MRI abnormalities, whether or not a child develops neurocognitive and behavioral impairments, is also dependent on other factors. Such other factors may include the presence of a stimulating home and/or school environment, educational level of the parents, and therapy use [23, 24].

Other considerations relevant to deciding on the use of MRI for the prediction of developmental outcomes are the substantial health care costs associated with its use. In many neonatal units, MRI technology is unavailable or its use is severely restricted. Also, expert neuroradiologists are needed for proper interpretation of the MRI results. In view of its potential prognostic capacity, it is therefore still debatable whether performing a standard term MRI is cost-effective.

### Limitations

This meta-analysis has some limitations that need to be considered. Although a considerable number of studies were identified on the subject, only a limited number of data points were available for each specific combination of MRI findings and neonatal outcome. Although even the results of only two studies can be pooled, the limited number of data points and often limited sample size per study imply limited power (hence wide confidence intervals) [25].

Also, the presence of heterogeneity may raise the question whether pooling of results is justified in our study. In prognostic meta-analysis, two possible reasons for heterogeneity of the data are known i.e., *clinical* heterogeneity, due to differences in features of the cohorts, and heterogeneity due to *threshold effect*. We estimate a smaller impact of the clinical heterogeneity as all cohorts included consecutive and comparable populations (although inadequate and inconsistent reporting of possible confounders in the studies, e.g., use of medication, birth weight, and presence of neonatal complications during admission, made it impossible to correct for potential confounders in our meta-analysis). Heterogeneity due to threshold effect is a common occurrence in many diagnostic test systematic reviews and probably explaining most of the heterogeneity in our meta-analysis [9]. The threshold effect in MRI tests is explained by the relative subjectivity of interpretations of MRI results e.g., one lesion on the MRI might be seen as abnormal for one radiologist but not by another. Also the use of different scoring systems and differences in background of the evaluators (neonatologists or radiologist) contribute to this type of heterogeneity. For this review, heterogeneity due to different scoring systems is probably the case in studies describing “brain abnormalities.” These studies not only include WMA as one of the MRI findings but also a composite of other MRI findings (i.e., IVH and/or increased ventricle size). However, since this heterogeneous definition of “brain abnormality” reflects common practice, we included these diverging MRI findings.

Furthermore, the quality of the included studies varied. In general, the majority of the studies were of good quality, although the lack of reporting of blinding of the MRI test at follow-up assessment in almost 50 % of the papers is a point of concern. However, in view of the limited number of included studies, subgroup analyses by excluding low quality studies is unlikely to resolve this question, as it would merely lead to broader confidence intervals [26]. As with all reviews, this systematic review is susceptible to publication bias. Especially cohort studies that did not show any predictive value of MRI have a lower chance of being published. The effect of publication bias may have resulted to overestimation of the predictive value of MRI in our meta-analysis.

### Recommendations for clinical care and further research

There is a solid evidence that very preterm birth and low birth weight has negative consequences on motor, neurocognitive, and behavioral functioning [1, 27, 28]. Preterm birth is also associated with variable degrees of brain injury and reduced brain volumes [18, 29]. A multitude of possible confounding factors play a role in the developmental outcomes of these fragile infants. Although MRI results can add valuable information on the prediction of long-term development, this information is in our opinion too marginal to use it on its own. A next step to consider is the performance of an individual patient data (IPD) analysis gathering the results from the individual level. First, this will enhance correction of confounders of the different cohort studies. Second, this extensive data-analyses technique may be used to develop a prognostic model, in which the presence of WMA on MRI can be combined with other biomarkers known to influence long-term development such as gender, neonatal history, clinical symptoms as infection [30], poor nutrition [31], use of steroids [32], low birth weight, socio-demographic factors, other imaging techniques as ultrasonography [33], or other promising MRI techniques that might show moderate prognostic accuracy in the near future (e.g., MR spectroscopy, diffusion tensor imaging (DTI), and neurite orientation dispersion and density imaging (NODDI)) [34]. A model statistically combining various relevant prognostic factors likely increases the accuracy to predict outcomes and may therefore be a more valuable tool for clinical use than MRI on its own.

## Conclusions

This meta-analysis shows that the presence of moderate/severe WMA on MRI around term equivalent age can predict CP and motor function in very preterm or low-birth-weight neonates with moderate sensitivity and specificity. The ability to predict other long-term outcomes such as neurocognitive and behavioral impairments is limited. Before considering the use of this test as a standard test in clinical practice, we encourage the continued use of routine MRI in a research setting to generate further evidence on its prognostic capacity together with other prognostic factors.

## Additional files

Additional file 1:
**Full search. Full search in Central, Medline, Embase and PsycInfo database.**
Additional file 2:
**Modified version of QUADAS-2 assessment tool.** Modified version of QUADAS-2 assessment tool to evaluate the risk of bias. Additional file 3:
**Standardized data extraction form.**
Additional file 4: Table S1. Study characteristics. Study details of the 20 included studies for meta-analysis.

## Abbreviations

BA: brain abnormality
CP: cerebral palsy
DEHSI: diffuse excessive high signal intensity
MRI: magnetic resonance imaging
sROC: summary receiver operating characteristic
WMA: white matter abnormalities
