# Supplementary material for: Predicting developmental outcomes in premature infants by term equivalent MRI: systematic review and meta-analysis
Source: Syst Rev. 2015 May 17;4:71. doi: 10.1186/s13643-015-0058-7 (PMC4438620; doi:10.1186/s13643-015-0058-7)
Supplement: Additional file 1: — Full search. Full search in Central, Medline, Embase and PsycInfo database. [file 13643_2015_58_MOESM1_ESM.doc]

**Appendix A: search**

**CENTRAL** (cochrane library)

#1 mri near/3 (neonat* or a term or term)

#2 (magnetic or resonance or imaging or spectroscopy or tensor or diffusion) near/5 (neonat* or newborn* or a term or term)

#3 volumetric mr imaging or fractional anisotropy or fluid attenuated inversion recovery or flair or apparent diffusion coefficient or diffuse excessive high signal intensity or dehsi or (diffusion near/4 imaging)

#4 (#1 or #2 or #3)

#5 MeSH descriptor: [Infant, Premature] explode all trees

#6 MeSH descriptor: [Intensive Care, Neonatal] explode all trees

#7 (birth weight near/4 week*) or (gestational age near/4 32 week*) or lower gestational age

#8 preterm* or prematur* or elbw or vlbw or low birth weight* or small for date or nicu

#9 #5 or #6 or #7 or #8

#10 #4 and #9

**MEDLINE**

1. (birth weight adj4 week*).tw.

2. ((gestational age adj4 32 week*) or lower gestational age).tw.

3. exp infant, low birth weight/ or infant, premature/ or neonatal intensive care/

4. (preterm* or prematur* or elbw or vlbw or low birth weight* or small for date or nicu).tw.

5. or/1-4

6. (mri adj4 (neonat* or newborn* or a term or a-term or term)).tw.

7. ((magnetic or resonance or imaging) adj5 (neonat* or newborn* or a term or term)).tw.

8. (volumetric mr imaging or fluid attenuated inversion recovery or flair or apparent diffusion coefficient or fractional anisotropy or diffuse excessive high signal intensity or dehsi or (diffusion adj4 imaging)).tw.

9. 6 or 7 or 8

10. (cohort or prospective or retrospective or longitudinal or prognosis or risk or case control or long term or longterm).tw.

11. exp cohort studies/ or exp prognosis/ or exp risk/ or case control studies/

12. 10 or 11

13. exp mental disorders diagnosed in childhood/

14. exp Nervous System Diseases/

15. exp mortality/

16. (corpus callosum or cerebrospinal fluid or white matter or grey matter or ((brain or cerebell*) adj2 (volum* or abnomalit* or atroph*))).tw.

17. (periventricular leu*omalacia or intraventricular h?emorrhage or cerebrospinal fluid or cerebellum).tw.

18. exp Cognition Disorders/ or cognition.tw.

19. (seizure* or epileps* or cerebral pals* or (learning adj3 disorder*) or deafness or blindness or (vision adj3 disorder*) or ((hearing adj3 disorder*) or visuospatial memory)).tw.

20. exp Intelligence Tests/

21. exp intelligence/

22. (intelligen* or stanford-binet or wechsler or bayley scal* or iq).tw.

23. exp Education, Special/

24. (outcome or neurological sequelae).mp.

25. (mental development index or psychomotor development index or social emotional development or movement assessment or executive function or neurodevelopment* or motor impairment or cognitive impairment or language skills or language development or language delay).tw.

26. or/13-25

27. 5 and 9 and 26

28. 5 and 9 and 12

29. 27 or 28

30. animal/ not (human/ and animal/)

31. 29 not 30

32. limit 31 to yr="1980 -Current"

**EMBASE** (1980- and weekly alerts)

1. (birth weight adj4 week*).tw.

2. ((gestational age adj4 32 week*) or lower gestational age).tw.

3. exp low birth weight/ or exp prematurity/ or neonatal intensive care.mp.

4. (preterm* or prematur* or elbw or vlbw or low birth weight* or small for date or nicu).tw.

5. or/1-4

6. (volumetric mr imaging or fractional anisotropy or fluid attenuated inversion recovery or flair or apparent diffusion coefficient or diffuse excessive high signal intensity or dehsi or (diffusion adj4 imaging)).tw.

7. (mri adj4 (neonat* or newborn* or a term or a-term or term)).tw.

8. ((magnetic or resonance or imaging) adj5 (neonat* or newborn* or a term or term)).tw.

9. 6 or 7 or 8

10. (cohort or prospective or retrospective or longitudinal or prognosis or risk or case control).tw.

11. cohort analyse/ or follow up/ or prospective study/ or retrospective study/ or exp prognosis/ or exp risk/ or case control study/

12. 10 or 11

13. exp mental disease/

14. exp Neurologic disease/

15. exp mortality/

16. (seizure* or epileps* or cerebral pals* or white matter or grey matter or ((brain or cerebell*) adj2 (volum* or abnomalit* or atroph*))).tw.

17. (periventricular leu*omalacia or intraventricular h?emorrhage or cerebrospinal fluid or cerebellum).tw.

18. cognitive defect/ or cognition.tw.

19. ((learning adj3 disorder*) or deafness or blindness or (vision adj3 disorder*) or ((hearing adj3 disorder*) or visuospatial memory)).tw.

20. exp Intelligence Test/

21. exp intelligence/

22. (intelligen* or stanford-binet or wechsler or bayley scal* or iq).tw.

23. exp special education/

24. (outcome or neurological sequelae).mp.

25. (mental development or psychomotor development or social emotional development or movement assessment or executive function or neurodevelopment* or motor impairment or cognitive impairment or language skills or language development or language delay).tw.

26. or/13-25

27. 5 and 9 and 26

28. 5 and 9 and 12

29. 27 or 28

30. (animal/ or nonhuman/) not (human/ or ((animal/ or nonhuman/) and human/))

31. 29 not 30

PsycInfo

1. (birth weight adj4 week*).tw,id.

2. ((gestational age adj4 32 week*) or lower gestational age).tw.

3. exp birth weight/ or premature birth/ or neonatal intensive care.mp,id.

4. (preterm* or prematur* or elbw or vlbw or low birth weight* or small for date or nicu).tw,id.

5. or/1-4

6. exp Magnetic Resonance Imaging/ or magnetic resonance spectroscopy.tw,id.

7. (mri or ((magnetic or cerebell*) adj3 imaging)).tw,id.

8. 6 or 7

9. neonatal period/ or newborn*.tw,id. or neonat*.tw,id.

10. 9 and 8

11. (mri adj4 (neonat* or newborn* or a term or a-term or term)).tw,id.

12. ((magnetic or resonance or imaging or spectroscopy or tensor or diffusion) adj5 (neonat* or newborn* or a term or term)).tw,id.

13. (volumetric mr imaging or fractional anisotropy or fluid attenuated inversion recovery or flair or apparent diffusion coefficient or diffuse excessive high signal intensity or dehsi or (diffusion adj4 imaging)).tw.

14. 11 or 12 or 13

15. 10 or 14

16. 5 and 15

17. (22* or 23* or 28* or 32*).cc.

18. (white matter or grey matter or cerebellum or ((brain or cerrebel*) adj2 (volum* or abnormalit* or atroph*)) or corpus callosum or periventricular leu*omalcia or intraventricular h?emorrhage or cerebrospinal fluid).mp,id.

19. 5 and 18

20. 5 and (8 or 13) and 18

21. 16 or 20

22. 17 and 21

23. limit 22 to yr="1980 -Current"
