# Supplementary material for: Predicting developmental outcomes in premature infants by term equivalent MRI: systematic review and meta-analysis
Source: Syst Rev. 2015 May 17;4:71. doi: 10.1186/s13643-015-0058-7 (PMC4438620; doi:10.1186/s13643-015-0058-7)
Supplement: Additional file 2: — Modified version of QUADAS-2 assessment tool. Modified version of QUADAS-2 assessment tool to evaluate the risk of bias. [file 13643_2015_58_MOESM2_ESM.doc]

**Scoring for methodologic quality: Risk of Bias and Applicability Judgement**

*Based on QUADAS-2*

Rater: **Main concerns Article:**

Author:

Date of publication:

Reference Manager Number:

*Patient Selection*

1. Describe methods of patient selection

1. Describe included patients (previous testing, presentation, intended use of MRI and setting)
2. Was a consecutive or random sample of patients enrolled? yes/no/unclear
3. Did the study avoid inappropriate exclusion? yes/no/unclear
4. Could the selection of patients have introduced bias? high/low/unclear
5. Are there concerns that the included patients do not match the review question? high/low/unclear

*MRI*

1. Describe how the MRI was conducted and interpreted
2. Were the MRI results interpreted without knowledge of the results of the

test at follow-up (*citerium test*) yes/no/unclear

1. If a threshold was used (on MRI findings) was it prespecified? yes/no/unclear
2. Could the conduct or interpretation of the MRI have introduced bias? high/low/unclear
3. Are there concerns that the MRI, its conduct or its interpretation differ

from the review question? high/low/unclear

*Test at follow-up (Criterium test)*

1. Describe the test at follow-up and how it was conducted and interpreted
2. Is the test at follow-up likely to correctly classify the target condition? yes/no/unclear
3. Were the test at follow-up results interpreted without knowledge of the results

of the MRI? yes/no/unclear

1. Could the test at follow-up, its conduct, or its interpretation have introduced bias? high/low/unclear
2. Are there concerns that the target condition as defined by the follow-up does

not match the review question? high/low/unclear

*Flow and Timing*

1. Describe any patients who did not receive the MRI or test-at follow-up (loss-to-follow-up) or who were excluded from the 2x2 table (refer to flow diagram)
2. Describe the interval and any interventions between MRI and the test at follow-up
3. Did all patients receive a test at follow-up? yes/no/unclear
   1. Percentage of loss-to follow up ____%
4. Did all patients receive the same test at follow-up? yes/no/unclear
5. Were all patients included in the analysis? yes/no/unclear
6. Could the patients flow have introduced bias? high/low/unclear
