# Supplementary material for: Predicting developmental outcomes in premature infants by term equivalent MRI: systematic review and meta-analysis
Source: Syst Rev. 2015 May 17;4:71. doi: 10.1186/s13643-015-0058-7 (PMC4438620; doi:10.1186/s13643-015-0058-7)
Supplement: Additional file 3: — Standardized data extraction form. [file 13643_2015_58_MOESM3_ESM.doc]

**Standardized data extraction form**

Rater:

Author:

Date of publication:

Reference Manager number:

**Characteristics of the study**

***Participants***

Total number of cases Total: MRI: Follow-up:

Number of preterms <32 weeks GA male female

Number of cases by GA

Important Baseline Characteristics

-Mean GA:

-Mean birth weight:

-Asphyxia

-Sepsis/NEC

-IUGR

-Interventions

-Other:

***MRI performance***

Gestational age of MRI performance

Used MRI technique

Prognostic factor (ex. White matter leasons/haemorrhage)

Used Classification for prognostic factor

Used cut-off values/range

Which part of the brain is imaged?

Cerebrum / Cerebellum

***Study design***

Prospective/Retrospective/Unclear

Single/Multi centre

***Test at follow-up***

Age at follow-up

Corrected?

Type of tests used

Cut-off points tests used

Which areas do these tests cover?

□ Neurologic performance

□ Behaviour

□ Cognitive

□ Somatic

Mortality numbers during follow-up?

**Results of the study**

***Main finding (related to prognostic factor)***

***2x2 table***

***Table 1: Used cut-off points MRI………………………………………………………………....***

***Used cut-off points Follow-up……………………………………………………………***

|  | ***Abnormal outcome (Follow-up)*** | ***Normal outcome***  ***(Follow-up)*** | ***Total*** |
| --- | --- | --- | --- |
| ***Test abnormal (MRI)*** |  |  |  |
| ***Test normal***  ***(MRI)*** |  |  |  |
|  |  |  |  |

***Table 2: Used cut-off points MRI………………………………………………………………....***

***Used cut-off points Follow-up……………………………………………………………***

|  | ***Abnormal outcome (Follow-up)*** | ***Normal outcome***  ***(Follow-up)*** | ***Total*** |
| --- | --- | --- | --- |
| ***Test abnormal (MRI)*** |  |  |  |
| ***Test normal***  ***(MRI)*** |  |  |  |
|  |  |  |  |

**EXTRA 2x2 Tables**

***Table 3: Used cut-off points MRI………………………………………………………………....***

***Used cut-off points Follow-up……………………………………………………………***

|  | ***Abnormal outcome (Follow-up)*** | ***Normal outcome***  ***(Follow-up)*** | ***Total*** |
| --- | --- | --- | --- |
| ***Test abnormal (MRI)*** |  |  |  |
| ***Test normal***  ***(MRI)*** |  |  |  |
|  |  |  |  |

***Table 4: Used cut-off points MRI………………………………………………………………....***

***Used cut-off points Follow-up……………………………………………………………***

|  | ***Abnormal outcome (Follow-up)*** | ***Normal outcome***  ***(Follow-up)*** | ***Total*** |
| --- | --- | --- | --- |
| ***Test abnormal (MRI)*** |  |  |  |
| ***Test normal***  ***(MRI)*** |  |  |  |
|  |  |  |  |

***Table 5: Used cut-off points MRI………………………………………………………………....***

***Used cut-off points Follow-up……………………………………………………………***

|  | ***Abnormal outcome (Follow-up)*** | ***Normal outcome***  ***(Follow-up)*** | ***Total*** |
| --- | --- | --- | --- |
| ***Test abnormal (MRI)*** |  |  |  |
| ***Test normal***  ***(MRI)*** |  |  |  |
|  |  |  |  |

***Table 6: Used cut-off points MRI………………………………………………………………....***

***Used cut-off points Follow-up……………………………………………………………***

|  | ***Abnormal outcome (Follow-up)*** | ***Normal outcome***  ***(Follow-up)*** | ***Total*** |
| --- | --- | --- | --- |
| ***Test abnormal (MRI)*** |  |  |  |
| ***Test normal***  ***(MRI)*** |  |  |  |
|  |  |  |  |
