# Supplementary material for: Predicting developmental outcomes in premature infants by term equivalent MRI: systematic review and meta-analysis
Source: Syst Rev. 2015 May 17;4:71. doi: 10.1186/s13643-015-0058-7 (PMC4438620; doi:10.1186/s13643-015-0058-7)
Supplement: Additional file 4: Table S1. — Study characteristics. Study details of the 20 included studies for meta-analysis. [file 13643_2015_58_MOESM4_ESM.doc]

**Table S1: Characteristics of studies included in Meta-analysis**

| **Study** | **Design** | **Country and Cohort** | **Male/**  **Female Ratio** | **MRI technique** | **Prognostic factor** | **Classification system** | **Defined Normal** | **Defined abnormal** | **Follow-up interval** | **Developmental outcome studied** | **Used Cut-off value for normal** | **Abnormal findings** |
| --- | --- | --- | --- | --- | --- | --- | --- | --- | --- | --- | --- | --- |
| Treyvaud et al., 2012 | Prospective single center | Australia, 2001-2003 | 87/79 | 1,5 Tesla, T1, T2, PD | WMA | Inder and Woodward^a,b^ | No WMA | Mild or Moderate-severe WMA | 2y corrected age | BSID-II MDI | No cut offs | Score <70* |
|  |  |  |  |  |  |  |  |  |  | BSID-II PDI | No cut offs | No cut offs |
|  |  |  |  |  |  |  |  |  |  | ITSEA | No cut offs | No cut offs |
| Kidokoro et al., 2011 | Prospective single center | Australia, 2001-2003 | 81/79 | 1,5 Tesla, T1, T2, PD | DEHSI, ADC values and FA | Own classification system | DEHSI grade 0 | DEHSI grade 1-4 | 2y corrected age | BSID-II MDI | Score >70 | Score <70 |
|  |  |  |  |  |  |  |  |  |  | BSID PDI | Score >70 | Score <70 |
|  |  |  |  |  |  |  |  |  |  | Neurological exam: CP | No signs of CP | Signs of CP |
| Spittle et al., 2011 | Prospective single center | Australia, 2001-2003 | 97/96 | 1,5 Tesla, T1, T2, PD | WMA | Inder and Woodward^a,b^ | 2 cut offs: nil and mild WMA | 2 cut offs: mild and moderate-severe WMA | 5y corrected age | MABC |  | Mild to severe <15th centile or moderate to severe <5th centile |
| Howard et al.,2011 | Prospective single center | Australia, 2001-2003 | 96/91 | 1,5 Tesla, T1, T2, PD | WMA | Inder and Woodward^a,b^ | 2 cut offs: nil and mild WMA | 2 cut offs: mild and moderate-severe WMA | 5y corrected age | KSEALS |  | Mild -1SD below the mean and severe -2SD below the mean |
| Beauchamp et al., 2008 | Prospective single center | Australia, 2001-2003 | 82/74 | 1,5 Tesla, T1, T2 | WMA, hippocampal volumes | Inder and Woodward^a,b^ | 2 cut offs: nil and mild WMA | 2 cut offs: mild and moderate-severe WMA | 2y corrected age | BSID-II MDI |  | Mild between -1 and -2SD and severe <-2SD |
|  |  |  |  |  |  |  |  |  |  | Delayed alternation/response task | Passing working memory task and/or 3 errors | Failing working memory training |
| Treyvaud et al, 2013 | Prospective single center | Australia, 2001-2003 | 94/83 | 1,5 Tesla, T1, T2, PD | WMA, GMA, CA | Own classification system | 2 cut offs: None and mild global brain abnormality | 2 cut offs: moderate and severe global brain abnormality | 7y corrected age | DAWBA | No psychiatric disorder | Psychiatric disorder |
| Munch et al., 2009 | Prospective single center | Finland 2001-2006 | 102/80 | 0.23 Tesla and 1,5 Tesla, T1, T2. | BA (IVH and WMA) | Own classification system | 2 cut offs: normal and minor brain pathology | 2 cut offs: minor and major brain pathology | 2y corrected age | BSID-II MDI | Score <-2SD (>70) | Score >-2SD (<70) |
|  |  |  |  |  |  |  |  |  |  | Neurological exam: CP | No signs of CP | Signs of CP |
|  |  |  |  |  |  |  |  |  |  | Hearing aids |  |  |
|  |  |  |  |  |  |  |  |  |  | NDI |  | Signs of CP and MDI <70 |
| Valkama et al., 2000 | Prospective single center | Finland 1993-1995 | 27/24 | 1,0 Tesla, T1, T2, PD | BA (parenchymal lesion) | Own classification system | Parenchymal lesions not present | Parenchymal lesions present | 18mo corrected age | Neurological exam: CP | No signs of CP | Signs of CP |
| Setanen et al., 2013 | Prospective single center | Finland 2001-2006 | 122/95 | 0,23 Tesla (n=125) and 1,5 Tesla. T1, T2 | BA (IVH, WMA and extracerebral space) | Own classification system | 2 cut offs: normal findings or ≥1 minor pathologies | 2 cut offs: one major pathology or several major pathologies | 5y chronological age | WPPSI-R (FSIQ) | Normal intelligence score ≥85 | Score 70-84 (>-1SD) or score <70 significantly below the normal intelligence |
|  |  |  |  |  |  |  |  |  |  | Hearing |  | Severe hearing impairment (amplification or hearing impairment >40dB) |
|  |  |  |  |  |  |  |  |  |  | Neurological exam: CP | No signs of CP | Signs of CP |
|  |  |  |  |  |  |  |  |  |  | NDI |  | FSIQ <85, CP, Severe hearing impairment (amplification or >40dB) or severe visual impairment (visual acuity <0.3 or blindness) |
| Gianni et al., 2007 | Prospective single center | Italy 1996-2001 | 71/70 | Conventional. Not further specified | BA (ventriculomegaly, cystic and noncystic PVL, focal parenchymal brain lesions) | No classification described | Normal | Abnormal: presence of major brain lesions | 36mo chronological age | GMDS | GQ>88 | GQ of <88 (1SD), Severe delay GQ<70 |
| Iwata et al., 2011 | Prospective single center | Japan, 1995-2001 | 46/30 | 0,5 Tesla T1, T2, FLAIR | DEHSI, WMA on FLAIR imaging, GMA | Inder and Woodward^a,b^ | Normal gray matter score | Abnormal grey matter score | 9y chronological age | WISC | IQ >85 | Mild IQ<85 or Moderate IQ<70 |
|  |  |  |  |  |  |  |  |  |  | WISC | IQ >85 | Mild IQ<85 or Moderate IQ<70 |
|  |  |  |  |  |  |  |  |  |  | Neurological exam: CP |  | Presence of hypertonicity, hyperreflexia, dystonia and spasticity |
|  |  |  |  |  |  |  |  |  |  | Parental interview: social emotional problems |  | Need for special assistance at school |
| Jeon et al, 2012 | Prospective single center | Korea 2004-2008 | 59/67 | 3 Tesla, T1, T2, FLAIR | DEHSI, WMA, IVH | Nanba^c^ and Papile et al^d^ | DEHSI:no, WMA:no | DEHSI: yes. WMA: cystic encephalomalacia, punctate lesions, loss of volume of white matter or corpus callosum, ventricular dilatation and myelination delay | 18-24mo corrected age | BSID-II MDI | Score <1SD (>85) | Mild between -1 and -2SD or severe >-2SD |
|  |  |  |  |  |  |  |  |  |  | BSID-II PDI | Score <1SD (>85) | Mild between -1 or -2SD and severe >-2SD |
|  |  |  |  |  |  |  |  |  |  | Neurological exam: CP | No signs of CP | signs of CP |
|  |  |  |  |  |  |  |  |  |  | Neurosensory impairment (hearing and vision) | Normal vision and hearing | Visual defect (requirement corrective lenses, surgery or strabismus or blindness) or hearing defect (hearing loss >30dB) |
| Clarc et al., 2010 | Prospective single center | New Zealand 1998-2000 | 55/52 | 1,5 Tesla, T1, T2, PD | WMA, GMA, brain volumes | Woodward et al^b^ | WMA and GMA 2 cut offs: nil and mild | 2 cut offs: mild an d moderate-severe WMA, mild and severe GMA | 6y corrected age | WPPSI-R verbal memory | Passing the memory task | Failing to pass the task |
|  |  |  |  |  |  |  |  |  |  | WPPSI-R visuospatial memory | Passing the memory task | Failing to pass the task |
| Woodward et al., 2006 | Prospective multi center | New Zealand 1998-2000 & Australia 2001-2002 | 78/88 | 1,5 Tesla, T1, T2, PD | WMA, GMA | Inder et al^a^ | 2 cut offs: nil and mild WMA and normal GMA | 2 cut offs:mild and moderate-severe WMA and abnormal GMA | 2y corrected age | BSID-II MDI | Score <1SD (>85) | Mild between -1 and -2SD or severe >-2SD |
|  |  |  |  |  |  |  |  |  |  | BSID-II PDI | Score <1SD (>85) | Mild between -1 and -2SD or severe >-2SD |
|  |  |  |  |  |  |  |  |  |  | Neurological exam: CP | No signs of CP | Signs of CP |
|  |  |  |  |  |  |  |  |  |  | Neurosensory impairment (hearing and vision) | Normal vision and hearing | Visual defect (requirement corrective lenses, surgery or strabismus or blindness) or hearing defect (hearing loss >30dB) |
|  |  |  |  |  |  |  |  |  |  | NDI |  | MDI or PDI <70, CP or neurosensory impairment |
| Woodward et al., 2012 | Prospective single center | New Zealand 1998-2000 | 54/52 | 1,5 Tesla, T1, T2, PD | WMA | Woodward et al^b^ | 2 cut offs: nil and mild WMA | 2 cut offs: mild and moderate-severe WMA | 4y corrected age | WPPSI-R |  | mild delay >-1SD or severe delay >-2SD |
|  |  |  |  |  |  |  |  |  |  | CELF-P |  |  |
|  |  |  |  |  |  |  |  |  |  | Composite of Executive function |  |  |
|  |  |  |  |  |  |  |  |  | 6y corrected age | WPPSI-R |  | mild delay >-1SD and severe delay >-2SD |
|  |  |  |  |  |  |  |  |  |  | WJ-III |  |  |
|  |  |  |  |  |  |  |  |  |  | Composite of Executive function |  |  |
| Skiöld et al., 2012 | Prospective single center | Sweden, 2004-2007 | 48/43 | 1,5 Tesla, T1, T2 | WMA, DEHSI | Inder et al^a^ | Normal and mild WMA, DEHSI not defined | Moderate-Severe WMA, DEHSI not defined | 30mo corrected age | BSID-III MDI |  | >-2SD (>70) |
|  |  |  |  |  |  |  |  |  |  | BSID-III PDI |  | >- 2SD (>70) |
|  |  |  |  |  |  |  |  |  |  | BSID-III language |  | >- 2SD (>70) |
|  |  |  |  |  |  |  |  |  |  | BSID-III Social Emotional |  | >- 2SD (>70) |
|  |  |  |  |  |  |  |  |  |  | Neurological exam: CP | Normal or unspecific sings | Abnormal (sings of CP) |
| de Bruine et al., 2011 | Prospective single center | The Netherlands 2006-2007 | 68/42 | 3 Tesla, T1, T2 | DEHSI, Punctate WM lesions and ventricular dilatation | Miller et al^e^ | DEHSI:no, WM lesions normal : ≤ 6 lesions. Normal ventricles: <12mm | DEHSI:yes. WM lesions abnormal : >6 lesions. Moderate dilatation ventrile 12-16mm, Severe >16mm | 2y corrected age | BSID-II MDI | Score <1SD (>85) | Mild between -1 and -2SD or severe >-2SD |
|  |  |  |  |  |  |  |  |  |  | BSID-II PDI | Score <1SD (>85) | Mild between -1 and -2SD or severe >-2SD |
|  |  |  |  |  |  |  |  |  |  | GMFCS Neurological exam: CP | GMFCS  score of 1 | Moderate CP score 2-3, Severe CP score of 4-5 |
| Rose et al., 2009 | Retrospective single center | USA 1999-2001 | 41/37 | 1,5 Tesla, T1, T2, FLAIR, DTI | BA (incl. ventriculomegaly and/or parenchymal abnormality), DWI | Own classification system and ADC values | continuous measures of ADC values |  | 18-22mo corrected age | BSID-II MDI |  | Score >-2SD (<70) |
|  |  |  |  |  |  |  |  |  |  | BSID-II PDI |  | very mild dysfluent gait to severe (no ambulation or independent sitting) motor impairment. |
|  |  |  |  |  |  |  |  |  |  | Neurological exam: CP |  |  |
|  |  |  |  |  |  |  |  |  |  | NDI (MDI+PDI <70 and CP) |  |  |
| Mirmiran et al., 2004 | Prospective single center | USA 1996-1999 | 31/30 | 1,5 Tesla, T1, T2, PD | WMA, heamorrhages, mineralisation and ventricular size | own classification system | normal score of C0 and C1 | abnormal score of C2 and C3 | 21-31 mo corrected age | Neurological exam: CP. abnormal muscle tone/movement in at least 1 extremity | No CP | mild, moderate and severe CP |
| Augustine et al., 2008 | Retrospective single center | USA 2001-2003 | 17/19 | 1,5 Tesla, T1, T2, FLAIR, MRS | BA (hemorrhage or mineratlization, ventriculomegly, parenchymal abnormality) | Own classifiction system | normal score C0 and C1 | abnormal score of C2 and C3 | 18-24mo corrected age | BSID-II MDI | Score <1SD (>85) | Score >1SD (<85) |
|  |  |  |  |  |  |  |  |  |  | BSID-II PDI | Score <1SD (>85) | Score >1SD (<85) |
|  |  |  |  |  |  |  |  |  |  | Neurological exam: CP. abnormal muscle tone/movement in at least 1 extremity | No CP | Signs of CP |

*data derived after contact with author.

ADC, apparent diffusion coefficient; BA, brain abnormality; BSID-II, bayley scales of infant development; CA, cerebellar abnormalities; CP, cerebral palsy; CELF-P, clinical evaluation of language fundamental; CVLT-C, california verbal learning test, children’s version; DAWBA, developmental and well-being assessment; DEHSI, diffuse excessive high signal intensity; DTI, diffusion tensor imaging; FA, fractional anisotropy; FLAIR, fluid attenuated inversion recovery imaging; FSIQ, full scale intelligence quotient; GMA, gray matter abnormality; GMDS, griffiths mental developmental scales; GMFCS, gross motor function classification system; GQ, general quotient, IVH, intraventricular haemorrhage; ITSEA, infant-toddler social and emotional assessment; KSEALS, kaufman survey of early academic and language skills; MABC, motor assessment battery for children; MDI, mental development index; MRS, magnetic resonance spectroscopy; MSML, multisearch multilocation search task; NDI, neurodevelopmental impairment; NEPSY II: neuropsychological assessment; PD, proton density; PDI, psychomotor development index; PVL, periventricular leukomalacia; SDQ, strengths and difficulties questionnaire; TBV, total brain volume; WISC: wechsler intelligence scale for children; WJ-III, woodcock johnson-III test tests of achievement; WMA, white matter abnormality; WPPSI, wechsler preschool and primary scale of intelligence; WPPSI-R: wechsler preschool and primary scale of intelligence-revised;

^a^ *Inder TE, Wells SJ, Mogridge NB, Spencer C, Volpe JJ. Defining the nature of the cerebral abnormalities in the premature infant: a qualitative magnetic resonance imaging study. J Pediatr 2003 Aug;143(2):171-9.*

*^b^ Woodward LJ, Anderson PJ, Austin NC, Howard K, Inder TE. Neonatal MRI to predict neurodevelopmental outcomes in preterm infants. N Engl J Med 2006 Aug 17;355:(7):685-94.*

^c^ *Nanba Y, Matsui K, Aida N, Sato Y, Toyoshima K, Kawataki M, et al. Magnetic resonance imaging regional T1 abnormalities at term accurately predict motor outcome in preterm infants. Pediatrics 2007 Jul;120(1):e10-e19.*

*^d^ Papile LA, Burstein J, Burstein R, Koffler H. Incidence and evolution of subependymal and intraventricular hemorrhage: a study of infants with birth weights less than 1,500 gm. J Pediatr 1978 Apr;92(4):529-34.*

^e^ *Miller SP, Cozzio CC, Goldstein RB, Ferriero DM, Partridge JC, Vigneron DB, et al. Comparing the diagnosis of white matter injury in premature newborns with serial MR imaging and transfontanel ultrasonography findings. AJNR Am J Neuroradiol 2003 Sep;24(8):1661-9.*
